# Sulbactam and Colistin Susceptibility Pattern Among Multidrug-Resistant Acinetobacter Isolates From Respiratory Samples

**DOI:** 10.7759/cureus.21802

**Published:** 2022-02-01

**Authors:** Mallika Sengupta, Sayantan Banerjee

**Affiliations:** 1 Microbiology, All India Institute of Medical Sciences, Kalyani, Kalyani, IND

**Keywords:** mic, colistin, sulbactam, mdr, acinetobacter, lrti

## Abstract

Background

*Acinetobacter* species are known to be important hospital-acquired pathogens. Unfortunately, multidrug-resistant *Acinetobacter *spp. has very limited options for an effective treatment.

Aim

To identify the common pathogens causing lower respiratory tract infections (LRTI), their antimicrobial susceptibility pattern, and determine the minimum inhibitory concentration (MIC) of sulbactam and colistin for *Acinetobacter *spp.

Materials and methods

A prospective study was done for a period of six months in a tertiary care hospital in Eastern India. The organisms causing LRTI were identified by conventional biochemical techniques and VITEK 2 Compact System (bioMérieux Inc., France). Antimicrobial susceptibility testing was performed using the Kirby‑Bauer disc diffusion method. MIC was also measured for *Acinetobacter *spp. to confirm certain antimicrobial agents using E-strips and micro broth dilution techniques.

Results

A total of 542 sputum and endotracheal tube aspirate (ETA) samples were examined during the study period. Among these, 109 samples showed growth of significant colony count of one or two organisms, yielding a sum of 115 isolates. Among these, there were 51 (44.35%) isolates of *Klebsiella pneumoniae*, 32 (27.83%) isolates of *Pseudomonas* spp., 30 (26.09%) isolates of *Acinetobacter* spp., and two (1.74%) isolates of *Stenotrophomonas maltophilia*. Although they were susceptible to colistin, *Acinetobacter* spp. was highly resistant to sulbactam.

Conclusion

Although colistin susceptibility was noted, the common pathogens causing LRTI were highly resistant to most drugs. Therefore, the causative organisms of LRTI and their susceptibility pattern should be determined to manage these cases effectively.

## Introduction

Lower respiratory tract infection (LRTI) is one of the most important and common health problems causing significant morbidity and mortality [1], accounting for 20-30% of all infections observed in a hospital. It is characterized by the high mortality in hospitalized patients [2]. The most common bacteria causing LRTIs in the intensive care unit (ICU) are *Pseudomonas, Acinetobacter, Klebsiella, Citrobacter, and Escherichia coli.* Several factors such as the standard of healthcare facilities available, characteristics of the at-risk population, immunosuppressive drugs, inappropriate antibiotic therapy, causative agent distribution, and antimicrobial resistance prevalence determine the incidence and associated mortality due to LRTI [3].

*Acinetobacter baumannii* is a major cause of healthcare-associated infections, particularly associated with hospital-acquired pneumonia, bloodstream infections, and septicemia in immunocompromised and moribund patients with severe underlying diseases. Over the last 20 years, a definite increase in multidrug resistance (MDR) rates to most antimicrobial agents with actions against *A. baumannii* has been observed [4]. The majority of the common non-fermenting Gram-negative bacilli (NFGNB) causing nosocomial infections are intrinsically resistant to multiple antibiotics and become resistant easily even to those antimicrobials to which they are susceptible. Therefore, NFGNB infections are difficult to treat. Carbapenems are the ideal treatment choice for these infections. However, the resistance to carbapenems has recently increased in these bacteria [5]. Hence, only very few agents are available to treat infections caused by these resistant non-fermenting pathogens. Colistin and polymyxin B can treat infections caused by these multidrug-resistant (MDR) pathogens. However, most of these drugs are associated with severe side effects, such as nephrotoxicity and neurotoxicity [6]. Sulbactam is an alternative which is used with other effective antibiotics for the treatment of MDR *Acinetobacter baumannii* infections [7].

In the last few years, increased antibiotic resistance has affected the selection of empirical treatment for LRTI. An effective knowledge of the likely prevalent strains with their antimicrobial resistance pattern will help in better patients care and in developing the antibiotic policy, that is antimicrobial stewardship. *Acinetobacter baumannii *is the most common species to have developed resistance to antibiotics. Due to increasing drug resistance, the available therapeutic agents are limited.

This study was conducted in a tertiary care hospital to track the resistance rate among the causative agents of LRTI. The minimum inhibitory concentration of sulbactam and colistin to Acinetobacter was also determined in this study.

## Materials and methods

A prospective study was conducted from November 2015 to April 2016 (6 months) in the Department of Microbiology in a 380-bed capacity tertiary care hospital, a medical college situated in Eastern India. The study was carried out after obtaining ethical clearance from the Institutional Review Board. Sputum and endotracheal tube aspirate (ETA) samples were collected from those clinically suspected of having LRTI in the inpatient, ICU, and outpatient departments.

A well-coughed out sputum sample or endotracheal aspirate from intubated patients was collected to determine the causative agents of LRTI. The samples were processed within 2 hours after the collection. Direct microscopy of the sample was performed to look for pus cells, epithelial cells, and bacteria. The sputum and ETA samples were streaked on blood, chocolate, and MacConkey agars and incubated at 37°C in the presence of carbon dioxide up to 48 hours. The growth of organisms and the number of colonies were observed (mild, moderate, or heavy growth). The isolates obtained from samples with significant pus cells and less epithelial cells [8] in the background of relevant supportive clinical features of LRTI as described in the standard guidelines were only included in the study.

The conventional biochemical tests such as catalase, oxidase, nitrate reduction, glucose fermentation, mannitol, lactose, indole production, urease, lysine decarboxylation, arginine decarboxylation and ornithine decarboxylation, methyl red test, Voges-Proskauer test, and VITEK 2 Compact System (BioMérieux Inc., France) were used to identify the organisms isolated.

The antimicrobial susceptibility was performed for these isolates on Mueller-Hinton agar plates by Kirby-Bauer disk diffusion method, and interpretation was performed following the Clinical and Laboratory Standards Institute guidelines M100-S25 version published in 2015 [9].

For *Enterobacteriaceae,* the isolates were tested against ceftriaxone (30 µg), cefotaxime (30 µg), cefepime (30 µg), amoxicillin-clavulanate (20/10 µg), piperacillin/tazobactam (100/10 µg), cefoperazone/sulbactam (75/30 µg), ciprofloxacin (5 µg), levofloxacin (5 µg), trimethoprim-sulphamethoxazole (1.25/23.75 µg), doxycycline (30 µg), amikacin (30 µg), gentamicin (10 µg), meropenem (10 µg), imipenem (10 µg), and colistin (10 µg).

For NFGNB including *Pseudomonas *spp., the isolates were tested against ceftazidime (30 µg), cefepime (30 µg), piperacillin/tazobactam (100/10 µg), cefoperazone/sulbactam (75/30 µg), ciprofloxacin (5 µg), levofloxacin (5 µg), amikacin (30 µg), gentamicin (10 µg), tobramycin (10 µg), trimethoprim-sulphamethoxazole (1.25/23.75 µg), doxycycline (30 µg), meropenem (10 µg), imipenem (10 µg), and colistin (10 µg). For *Pseudomonas* spp., aztreonam (30 µg) was also included.

For* Acinetobacter* isolates, minimum inhibitory concentration (MIC) testing was performed for sulbactam and colistin using E-test and micro broth dilution technique for confirmation.

MDRs are the organisms resistant to any three different classes of antibiotics [10]. To detect the extended-spectrum beta-lactamase (ESBL) producers among *Enterobacteriaceae*, a disk diffusion method with ceftriaxone and cefotaxime was performed for screening, and simultaneous testing with cefotaxime (30 μg) and combination of cefotaxime/clavulanate (30/10 μg) through the disk diffusion method was used for confirmation (9).

All data were entered in the Excel spreadsheet. Data were summarized using mean with standard deviation for continuous variables, and frequency with percentages for categorical variables. A chi-squared test was used to evaluate the association of categorical variables, and a p-value <0.05 was considered significant.

## Results

A total of 542 sputum and endotracheal tube aspirate samples were received, fulfilling the inclusion criteria. Among these, 109 (20.11%) samples showed growth of significant colony count of one or two organisms, yielding a sum of 115 isolates fulfilling the criteria for significance.

Of these 109 patients with significant growth of organisms, 83 (76.15%) were male, and 26 (23.85%) were female, showing a ratio of 3.2:1. The age of the patients was between 14 years and 92 years (mean = 48.3 years, SD = 12.4).

Among the positive samples, two (1.83%) were from OPD, 58 (53.21%) from inpatient wards, and 49 (44.95%) from the ICU. In addition, there were 70 (64.22%) samples from the chest medicine department and 39 (35.78%) samples from the medicine department. These 109 samples showed growth of 115 isolates, of which the outpatient and ward samples had single pathogen isolates, whereas few ICU samples showed growth of two pathogenic organisms. Among the 115 isolates, there were 51 (44.35%) isolates of *Klebsiella pneumoniae*, 32 (27.83%) isolates of *Pseudomonas* spp., 30 (26.09%) isolates of *Acinetobacter* spp., and two (1.74%) isolates of *Stenotrophomonas maltophilia* (Table 1).

**Table 1:** Distribution of organisms from the outpatient, inpatient wards, and ICU.

Organism	OPD	inpatient Ward	ICU	Total
Klebsiella pneumoniae	2	30	19	51
Pseudomonas spp.	0	18	14	32
Acinetobacter spp.	0	8	22	30
Stenotrophomonas maltophilia	0	2	0	2
Total	2	58	55	115

Among the 51 isolates of *Klebsiella pneumoniae*, most were resistant to cephalosporins and fluoroquinolones. More than 50% of the isolates were resistant to imipenem and meropenem. All 51 (100%) were susceptible to colistin. The majority of the isolates (66.67%) were susceptible to doxycycline (Table 2). There were 33 (64.70%) multidrug-resistant and 46 (90.2%) were ESBL-producer isolates of *Klebsiella pneumoniae*.

**Table 2:** Susceptibility of Klebsiella pneumoniae (n = 51) to different antibiotics.

Antibiotic	Susceptible	Intermediate susceptible	Resistant
Ceftriaxone	5 (9.8%)	-	46 (90.2%)
Cefotaxime	5 (9.8%)	-	46 (90.2%)
Cefepime	7 (13.73%)	-	44 (86.27%)
Amoxicillin-clavulanate	3 (5.88%)	-	48 (94.12%)
Piperacillin-tazobactam	11 (21.57%)	1 (1.96%)	39 (76.47%)
Cefoperazone-sulbactam	15 (29.41%)	-	36 (70.59%)
Ciprofloxacin	10 (19.61%)	2 (3.92%)	39 (76.47%)
Levofloxacin	14 (27.45%)	3 (5.88%)	34 (66.67%)
Trimethoprim-sulfamethoxazole	18 (35.29%)	3 (5.88%)	30 (58.83%)
Doxycycline	34 (66.67%)	-	17 (33.33%)
Amikacin	18 (35.29%)	-	33 (64.71%)
Gentamicin	18 (35.29%)	-	33 (64.71%)
Meropenem	20 (39.22%)	-	31 (60.78%)
Imipenem	22 (43.14%)	2 (3.92%)	27 (52.94%)
Colistin	51 (100%)	-	0 (0%)

Among the 32 isolates of* Pseudomonas* spp., there were 31 isolates of *Pseudomonas aeruginosa* and one isolate of* Pseudomonas putida*. All were susceptible to colistin and polymyxin B. In addition, the isolates were highly resistant to ceftazidime (Table 3). A total of 15 (46.87%) isolates of *Pseudomonas* were multidrug-resistant or resistant to three different classes of antibiotics.

**Table 3:** Susceptibility of Pseudomonas spp. (n = 32) to different antibiotics.

Antibiotic	Susceptible	Intermediate susceptible	Resistance
Ceftazidime	1 (3.12%)	-	31 (96.88%)
Cefepime	12 (37.5%)	-	20 (62.5%)
Aztreonam	14 (43.75%)	5 (15.62%)	13 (40.63%)
Piperacillin- tazobactam	17 (53.13%)	-	15 (46.87%)
Cefoperazone-sulbactam	20 (62.5%)	-	12 (37.5%)
Ciprofloxacin	12 (37.5%)	-	20 (62.5%)
Levofloxacin	14 (43.75%)	-	18 (56.25%)
Amikacin	17 (53.13%)	-	15 (46.87%)
Gentamicin	17 (53.13%)	-	15 (46.87%)
Tobramycin	17 (53.13%)	-	15 (46.87%)
Meropenem	14 (43.75%)	-	18 (56.25%)
Imipenem	14 (43.75%)	-	18 (56.25%)
Colistin	32 (100%)	-	0 (0%)

Among the 30 isolates of *Acinetobacter* spp., all were completely resistant to ceftazidime, cefepime, piperacillin/tazobactam, cefoperazone/ sulbactam, ciprofloxacin, amikacin, gentamicin, tobramycin, meropenem, and imipenem. However, all isolates were susceptible to colistin (Table 4).

**Table 4:** Susceptibility of Acinetobacter spp. (n = 30) to different antibiotics.

Antibiotic	Susceptible	Intermediate susceptible	Resistance
Ceftazidime	0 (0%)	-	30 (100%)
Cefepime	0 (0%)	-	30 (100%)
Piperacillin- tazobactam	0 (0%)	-	30 (100%)
Cefoperazone-sulbactam	0 (0%)	-	30 (100%)
Ciprofloxacin	0 (0%)	-	30 (100%)
Levofloxacin	1 (3.33%)	1 (3.33%)	28 (93.34%)
Amikacin	0 (0%)	-	30 (100%)
Gentamicin	0 (0%)	-	30 (100%)
Tobramycin	0 (0%)	-	30 (100%)
Meropenem	0 (0%)	-	30 (100%)
Imipenem	0 (0%)	-	30 (100%)
Trimethoprim-sulfamethoxazole	1 (3.33%)	2 (6.67%)	27 (90%)
Doxycycline	3 (10%)		27 (90%)
Colistin	30 (100%)	-	0

The MIC was performed for sulbactam and colistin. All* Acinetobacter* were found to be resistant to sulbactam and sensitive to colistin (Table 5). In addition, all Acinetobacter isolates were multidrug-resistant.

**Table 5:** The MIC of Acinetobacter spp. MIC: Minimum inhibitory concentration.

Antibiotic	Range	MIC50	MIC90
Sulbactam	64–256 µg/ ml	128 µg/ ml	256 µg/ml
Colistin	0.5–2 µg/ ml	1 µg/ml	2 µg/ml

The two isolates of* Stenotrophomonas maltophilia* were susceptible to levofloxacin and trimethoprim-sulfamethoxazole.

## Discussion

In this prospective study conducted for six months in a tertiary care medical college in India, the common pathogens causing LRTI were *K. pneumoniae* in 44.35% cases, *Pseudomonas *spp. in 27.83%, *Acinetobacter *spp. in 26.09%, and *Stenotrophomonas maltophilia* in two cases. This is similar to the findings of a study performed in Nigeria among 954 sputum samples, where 431 (45.2%) were positive for microorganisms. A single, unique pathogen was recovered in 415 patients (96.3%), and 16 (3.7%) were polymicrobial. The most predominant single pathogen was *K. pneumoniae* in 49.9% [1]. However, in our study, 20.11% of samples showed growth of one or more pathogens. In a study in India, out of 161 isolates from LRTIs, 154 (95.6%) were Gram-negative bacilli, including* P. aeruginosa *(35%), *A. baumannii *(23.6%), and *K. pneumoniae* (13.6%) [11]. Similarly, among 28 mechanically ventilated patients, 82% were found to have developed LRTI. Aerobic Gram-negative bacilli accounted for 79% of infections, *Klebsiella* was responsible for 39%, whereas *Pseudomonas *and Escherichia accounted for 18% each, and 4% were caused by *Acinetobacter* [12].

In this study, two samples were obtained from the OPD, 58 from inpatient wards, and 49 from the ICU. However, out of 30 *Acinetobacter* isolates, 22 were from the ICU, and eight were from the ward. Maraki S et al. reported in 2016 from Greece that out of 914 clinical isolates, 493 *A. baumannii* samples were recovered from the ICU, 252 from the medical ward, and 169 from surgical wards [4].

In the current study, out of 109 patients with significant growth of organisms, 83 (76.15%) were males, and 26 (23.85%) were females. However, in a study of 200 LRTI cases, 66% were males, and 34% were females [13] because males are at greater risk of developing LRTI.

Among the 51 isolates of *K. pneumoniae*, 33 isolates were found to be MDR. There were 15 (46.87%) isolates of *Pseudomonas*, which were MDR. In a multicentric study by Claeys KC et al., 21.6% of *P. aeruginosa* isolates were MDR, 7.6% of which were extensively drug-resistant. Among the* A. baumannii*, 64.4% were identified as MDR, and 16.3% were extensively drug-resistant [14]. Dong L et al. found that 42.2% of *K. pneumoniae* isolated from patients with acute LRTI in China were ESBL-positive strains [15]. However, 90% of* K. pneumoniae* were ESBL producers in this study. Among the 243 Gram-negative bacteria, 89 (36.62%) were ESBL isolates [16].

Maraki S et al. found that only 4.9% of *Acinetobacter* isolates were fully susceptible to the antimicrobials tested, whereas 92.89% of them were MDR or resistant to ≥3 different classes of antibiotics. The most resistant isolates were obtained from the ICU, followed by the surgical and medical wards. The most effective antimicrobial agents were colistin, amikacin, trimethoprim/sulfamethoxazole, tigecycline, and tobramycin in descending order. Nevertheless, except for colistin, no antibiotic was associated with a susceptibility rate >40% during the entire study period [4]. In this study, all *Acinetobacter *isolates were MDR and were only susceptible to colistin. Both were tested using disc diffusion and MIC methods. This is consistent with the study done by Lee H et al., who found that none of the extensively drug-resistant NFGNB was susceptible to any of the tested antibiotics, except colistin [5]. In another study by Gaur A et al., out of 265* Acinetobacter* spp. isolated from 1242 culture-positive samples from hospitalized patients, 91% were *A. baumannii* and 9% were *A. lwoffii*. On antimicrobial susceptibility testing, *Acinetobacter* spp. showed >80% resistance to third-generation cephalosporins. Among quinolones, 81% of the isolates were resistant to ciprofloxacin. Cefoperazone/sulbactam combination was effective with an overall resistance of 31% [17]. However, the current study showed that complete resistance of* Acinetobacter* spp. was noted on cefoperazone/sulbactam and even MIC of sulbactam.

In a study by Laishram S et al., the good bactericidal activity of 70-100% was noted with the combinations tested by synergy among sulbactam, meropenem, and colistin in carbapenem-resistant *K. pneumoniae* and *A. baumannii* [18]. In a meta-analysis on sulbactam-based therapy for *A. baumannii*, including four studies, sulbactam was given with ampicillin, carbapenem, or cefoperazone. Comparator drugs included colistin, cephalosporins, anti-pseudomonas penicillin, fluoroquinolones, minocycline/doxycycline, aminoglycosides, tigecycline, polymyxin, imipenem/cilastatin, and combination therapy. The combined clinical response rate odds ratio did not significantly favor sulbactam-based therapy over comparator therapy [19]. In this in vitro study, *Acinetobacter* isolates were found to be resistant to sulbactam.

In a study conducted in Poland for causative agents of pneumonia, most isolated pathogens included *A. baumannii* (35.8%), *Staphylococcus aureus* (27.6%), *K. pneumoniae* (19.4%), and *Pseudomonas aeruginosa* (16.2%). MDR Gram-negative bacteria exhibited 100% susceptibility to colistin only. Similarly, this study also found that MDR organisms were susceptible to colistin, including the *Acinetobacter* isolates.

## Conclusions

The limitations of this study were that genes responsible for resistant bacteria were beyond the scope of the study. Moreover, the therapeutic response of patients was not considered in this study.

Moreover, most pathogens for LRTI were MDR organisms with limited treatment options. It was also found that Acinetobacter spp. were not susceptible to sulbactam. Hence, proper culture and identification of pathogens causing LRTI are required with their susceptibility pattern for the management and prevention of resistance.
